# Stemness subtypes in lower-grade glioma with prognostic biomarkers, tumor microenvironment, and treatment response

**DOI:** 10.1038/s41598-024-65717-7

**Published:** 2024-06-26

**Authors:** Shengda Ye, Bin Yang, Liu Yang, Wei Wei, Mingyue Fu, Yu Yan, Bo Wang, Xiang Li, Chen Liang, Wenyuan Zhao

**Affiliations:** 1 Brain Research Center, Zhongnan Hospital of Wuhan University, Wuhan, China; 2 Department of Neurosurgery, Zhongnan Hospital of Wuhan University, Wuhan, China; 3 Department of Neurosurgery, Central Theater General Hospital of the Chinese People’s Liberation Army, Wuhan, China; 4 Frontier Science Center for Immunology and Metabolism, Wuhan, China; 5 Medical Research Institute, Wuhan University, Wuhan, China; 6 Sino-Italian Ascula Brain Science Joint Laboratory, Wuhan, China; 7 Department of Radiation and Medical Oncology, Zhongnan Hospital of Wuhan University, Wuhan, China; 8 Cancer Hospital of Zhongnan Hospital of Wuhan University, Wuhan, China; 9 Cancer Clinical Study Center of Hubei Province, Wuhan, China; 10 Hubei Key Laboratory of Tumor Biological Behavior, Wuhan, China

**Keywords:** Lower grade glioma, Stem cell, Tumor microenvironment, Bioinformatics, Nomogram, Cancer, Cell biology

## Abstract

Our research endeavors are directed towards unraveling the stem cell characteristics of lower-grade glioma patients, with the ultimate goal of formulating personalized treatment strategies. We computed enrichment stemness scores and performed consensus clustering to categorize phenotypes. Subsequently, we constructed a prognostic risk model using weighted gene correlation network analysis (WGCNA), random survival forest regression analysis as well as full subset regression analysis. To validate the expression differences of key genes, we employed experimental methods such as quantitative Polymerase Chain Reaction (qPCR) and assessed cell line proliferation, migration, and invasion. Three subtypes were assigned to patients diagnosed with LGG. Notably, Cluster 2 (C2), exhibiting the poorest survival outcomes, manifested characteristics indicative of the subtype characterized by immunosuppression. This was marked by elevated levels of M1 macrophages, activated mast cells, along with higher immune and stromal scores. Four hub genes—CDCA8, ORC1, DLGAP5, and SMC4—were identified and validated through cell experiments and qPCR. Subsequently, these validated genes were utilized to construct a stemness risk signature. Which revealed that Lower-Grade Glioma (LGG) patients with lower scores were more inclined to demonstrate favorable responses to immune therapy. Our study illuminates the stemness characteristics of gliomas, which lays the foundation for developing therapeutic approaches targeting CSCs and enhancing the efficacy of current immunotherapies. By identifying the stemness subtype and its correlation with prognosis and TME patterns in glioma patients, we aim to advance the development of personalized treatments, enhancing the ability to predict and improve overall patient prognosis.

## Introduction

Glioma stands out as one of the most prevalent intracranial tumors, presenting a formidable challenge in terms of treatment and cure. Lower-grade glioma, which is classified into two grades (grade II and grade III), accounts for 10–20% of all primary brain tumors^1^. While the outlook is considerably more favorable compared to high-grade gliomas, the median survival is only 5.6 to 13.3 years, depending on the type of pathology^2^. The goal of patients is often to extend overall survival with quality of life. The more favorable prognosis of lower-grade glioma compared to high-grade gliomas is uniquely challenging for both doctors and patients, requiring adjustments in the treatment approach to prevent tumor recurrence and high-level transformation^3^. Thus, individualized treatment is more needed in lower-grade gliomas.

CSCs are a subgroup of glioma cells that have the characteristics of unrestricted proliferation, multidirectional differentiation, and inducing tumor drug resistance^4^. Because CSCs have extensive proliferative capacity^5,6^, they can form diffuse metastatic tumors and affect the progression, metastasis, and treatment resistance of glioma^7^, making them a promising therapeutic target^8^.

In addition, the tumor microenvironment (TME) will continuously communicate with CSCs through intercellular crosstalk^9^. Several CSC biomarkers have been identified to contribute to the development, progression, and prognosis of glioma^10–12^. Research indicates that glioma-associated CSCs secrete higher levels of TGF-β compared to other types of tumors^13^. CSCs have the ability to release interleukin-6 (IL-6), triggering the activation of STAT3 signaling in tumor cells and promoting tumor growth^14^. CSCs also can exhibit elevated expression levels of the epidermal growth factor receptor, leading to increased proliferation and survival^15^. Therefore, exploring the interaction between CSCs with genetic heterogenicity and TME characteristics will help to determine the targeted treatment strategy for glioma, upgrade patient outcomes and extend survival periods.

To enhance our understanding of the complexities inherent in the stemness landscape of glioma, we performed a comprehensive analysis based on an extensive collection of shared sets comprising 26 genes related to stem cells. The single-sample gene set enrichment analysis (ssGSEA) was used to facilitate the evaluation of the stem cell phenotype through the expression of all genes extracted from a substantial number of glioma samples. Utilizing unsupervised clustering techniques, we identified three distinct stemness subtypes with varying degrees of stemness. Proceeding from this, we pinpointed genes exhibiting a high level of correlation with the aforementioned stemness subtypes, as well as prognosis, using WGCNA.

After conducting all subset regression analysis, and performing single-factor Cox analysis, we developed a stemness-related prognostic signature. Subsequently, we explored its associations with TME characteristics, prognosis, immunotherapy efficacy, and the effectiveness of chemotherapy in lower-grade glioma. Our study successfully established a stem cell risk scoring system, providing a valuable tool for evaluating the glioma stem cell landscape.

## Materials and methods

### LGG tissue specimen collection

Between December 2021 and June 2022, we obtained five LGG tissue specimens and six normal peritumoral tissue specimens from the Department of Neurosurgery at Zhongnan Hospital of Wuhan University. All enrolled patients had a verified LGG diagnosis through pathology, gave informed consent, and the study received ethical approval from the committee.

### LGG cohorts consolidation and preprocessing

We gathered 644 LGG samples from three different cohorts In aggregate, including TCGA-LGG, CGGA, and GSE43378 (grade II and grade III), along with their corresponding clinical and survival annotations. To ensure sample comparability, we acquired data for TCGA-LGG using the Cancer Genome Atlas (TCGA) database (https://genomecancer.ucsc.edu/). And transformed it into transcripts per million (TPM) form, accounting for differences in gene length^16^. As for the remaining five glioma-associated data cohorts, CGGA^17–20^, GSE43378^21^, Gravendeel^22^, GSE107850^23^ and Rembrandt^24^, we retrieved them from the GlioVis database (http://gliovis.bioinfo.cnio.es/)^25^ with the samples belonging to high-grade gliomas proposed. And preprocessed them via the Robust Multichip Average algorithm^26^. The method sva (3.44.0) was applied to mitigate the batch effect caused by different sequencing platforms, equipment, and reagents with the combat function^27^.

### Collection and grouping of stem cell characteristics of LGG stem cell subtypes

To comprehensively identify LGG stemness subtypes, we gathered an impressive compilation of 26 sets which includ stem cell-related genes from the highly esteemed Internet basic tool StemChecker, which is accessible at http://stemchecker.sysbiolab.eu/^28^. Subsequently, utilizing the GSVA (Gene Set Variation Analysis) R package (v1.44.5)^29^, all LGG sample’ enrichment scores of stem cell-related gene sets were calculated. This was achieved through the strategic implementation of the ssGSEA algorithm. As an integral next step in our study, the ssGSEA scores obtained were then subjected to unsupervised consensus clustering on LGG samples. This was accomplished via the ConsensusClusterPlus R package (v1.60.0)^30^, which was executed using the K-means clustering method based on Euclidean distance. In order to ensure optimal accuracy and reliability, the clustering analysis was repeated a staggering 1000 times. Ultimately, the consensusClusterPlus R package and factoextra R package^31^ were implemented in order to expertly and effectively determine the ideal number of clusters.

### Tumor microenvironment (TME) infiltration exploration

To thoroughly explore the intricacies of TME infiltrations in LGG samples, The well-known CIBERSORT deconvolution algorithm^32^ is utilized for assess the relative proportion of common immune cells based on the standardized large sample gene expression profile. With the "CIBERSORT" R package being utilized in conjunction with 1000 permutations and the gene signature of LM22 leukocyte. In this way, the TME fractions in each LGG sample were expertly and effectively quantified. In addition, a rigorous screening process was executed on samples with P < 0.05 as the cut-off of CIBERSORT analysis. In order to ensure maximal accuracy and dependability. Moreover, the incredibly powerful and highly regarded ESTIMATE algorithm^33^ was leveraged to estimate the stromal and immune scores for research dataset. This was accomplished through the R package estimate, the algorithm that can estimate the immune and mechanism level of the sample through RNA-seq data, and then evaluate the purity of the tumor.

### Predicting immunotherapy response and chemotherapeutic sensitivity

To predict chemosensitivity in LGG samples, we used oncoPredict (V0.2)^34^, an R package based on Genomics of Drug Sensitivity in Cancer (GDSC) (https://www.cancerRxgene.org/)^35^, to compute the number of half-maximum inhibitory concentration (IC50) of various chemotherapeutic agents using ridge regression^36^. To assess prediction accuracy, tenfold cross-validation was employed. Additionally, we estimated immunotherapeutic responses in LGG patients via Tumor Immune Dysfunction and Exclusion (TIDE), a network of scientific research tools (http://tide.dfci.harvard.edu/).

### WGCNA for key module identification

To construct an expression matrix from TCGA-LGG data, we used the GoodSamplesGenes method and sample network approach. We applied a statistical significance criteria value of Z.Ku = 2.5 (Z.ku = (ku-mean(k))/(sqrt(var(k))) that produced exceptional results^37^. Using the WGCNA R package (V1.71), we generated co-expression networks. Via the branch cutting approach, all genes were split into gene modules with min-ClusterSize = 30 and deepSplit = 2 set as crucial parameters^38^. We selected modules with high correlation (greater than 0.6) by assessing the variances in module eigengenes. To identify key modules associated with cluster (selected disease characteristics is cluster C1 or cluster C2), we evaluated gene significance (GS) and calculated module membership (MM) based on all genes’ value of average GS. Finally, we chose the module considered the most pertinent and crucial, as elucidated in the aforementioned description.

### Calculation of the mRNA stemness index (mRNAsi)

We use the OCLR algorithm based on machine learning of a class of logistic regression, which is trained with embryonic stem cells and their differentiated progenitor cells as data set samples, to evaluate the mRNAsi of each LGG sample, following the methodology proposed by Malta et al.^39^. This method is effective in predicting the stemness of cancer, and the OCLR approach has been previously used for this purpose.

### Development and validation of a prognostic stemness model

We used single Cox regression analysis (P < 0.05) to identify hub genes which were statistically associated with overall survival (OS) data in the TCGA dataset(TCGA-LGG, CGGA and gse43378)^40^. RandomForestSRC R package (v3.1.1) was utilized for the construction of random survival forest prognosis model to narrow the range of key genes^41^. The criterion for inclusion of genes in the next analysis is that the relative importance is greater than 0.25. After listing all the combinations, we obtained four genes through full subset regression with R package leaps (V3.1) and constructed a signature named stemness-risks core. The risk scores were calculated following the formula: stemness Risk score = , 'n' represents the number of genes in the signature and the value of Expi is the tpm expression profile values of the corresponding genes. We verified the accuracy of survival evaluation via the roc curve of stem cell-related risk score in MMD1(Gravendeel, GSE107850 and Rembrandt)^42^. We also drew the KM curve of three data sets in TCGA (TCGA-LGG, CGGA, GSE43378) separately. We also verified the protein levels of each gene were retrieved from the Human Protein Atlas (HPA) databasee^43^.

### Nomogram construction and verification

The nomogram was developed based on the prognostic stemness model, and cross-validation was performed to prevent overfitting^44^. The accuracy of the nomogram was assessed through the examination of a calibration curve., where the 45° line represented the highest degree of prediction potential. Validation of the feasibility and value of clinical applications of nomograms based on decision analysis curves, which was performed using the R package rmda (V1.6)^45,46^.

### Hub gene functional exploration

According to the difference in central gene expression, we conducted an enrichment analysis to determine the GO and KEGG pathway with significant differences and enrichment in the C1 and C2 groups of LGG. The c2.cp.kegg.v7.4.symbols.gmt gene set was annotated, and pathways were considered significantly enriched if they met the following criteria: a gene size (n) of 20%, FDR of 25%, |ES|> 6, and P < 0.05. The standard significantly enriched by KEGG and GO pathways was used in the analysis^47^. To investigate the differences in pathways and marker genomes differences between the lower and high stem cell related risk score groups of LGG patients, all 644 patients were first distinguished into two groups based on their stemness-risk scores, with the median value used as the threshold. Next, We conducted gene cluster enrichment analysis (GSEA) through the clusterProfiler R package^48^.

### Transcription-level expression validation by RT-qPCR

RT-qPCR is a molecular biology technique used to measure the expression level of RNA^49^. In this technique, RNA is initially reverse transcribed into complementary DNA (cDNA) through the action of the reverse transcriptase enzyme with the SuperRT RT reagent Kit (Vazyme, China), and then the cDNA is amplified using quantitative polymerase chain reaction (qPCR). Real-time monitoring of the amplification process is carried out using fluorescent dyes, enabling the quantification of the initial amount of RNA. The 2(-Ct) technique is commonly used to analyze the data generated from RT-qPCR, where Ct values are normalized to a housekeeping gene and expressed relative to a control sample. In this study, RNA was extracted from both LGG tumors and adjacent normal peritumoral tissues using RNAiso Plus (Takara, Japan). Correspondingly, the quality of RNA was evaluated before performing RT-qPCR using specific primers and commercial kits^50^. The primer sequence is shown in supplementary Table S1.

### Cell Culture

The HS683 human glioma cell line was sourced from Wuhan Procell Life Science and Technology Co., Ltd. (Procell, China). Cultivation involved the use of Dulbecco’s Modified Eagle’s Medium (DMEM, Servicebio, China) supplemented with 10% fetal bovine serum (FBS, Gibco, USA) and 1% antibiotics mix (Servicebio, China). Cultures were maintained at 5% CO2 and 37 °C, with regular passaging every 1–2 days to ensure exponential growth.

### Cell transfection

For transient transfection of siRNAs into glioma cells, the RNATransMate system (Sangon Biotech, China) was employed following the manufacturer’s instructions. The siRNAs were chemically synthesized by Sangon Biotech (China). The siRNA sequences for these genes are shown in supplementary Table S2.

### Lentiviral infection of glioma cells

We used a 6-well plate under a biosafety cabinet to seed well-conditioned HS683 cells in the logarithmic growth phase, and continuously cultured them in an incubator until the cell density reached approximately 50%, at which point the infection was performed. Fresh culture medium containing polybrene was prepared in advance. Then, the IDH R132H mutant lentivirus purchased from Heyuan company was used for the infection. At the beginning of the infection, the old medium in each well of the 6-well plate was aspirated, and then the prepared polybrene-containing medium was slowly added along the sidewall of each well. The plate was incubated horizontally in the incubator for 24 h. The next day, the medium was replaced with polybrene-free medium, and the plate was incubated horizontally in the incubator for another 48 h. Finally, the optimal concentration of puromycin was used to select the target cells. A small amount of the selected target cell samples was used for protein extraction, and qPCR was performed to detect the stable knockdown effect. After verification, the cells were expanded using puromycin-containing medium or used for subsequent experiments.

### Knockdown of target genes in mutant strains

Under a biosafety cabinet, seed the well-conditioned, selected IDH1 mutant HS683 cells into a 6-well plate. When the cell density reaches 50–60%, perform the transfection operation with the following system: Solution A consists of 250 μL Opti-MEM and 7.5 μL Lipomaster3000, while Solution B consists of 250 μL Opti-MEM and 4 μL siRNA. After preparation, gently mix with a pipette, add Solution B to Solution A dropwise, mix well, and let it stand at room temperature for 10 min before adding it to the 6-well plate. Replace the medium 24 h later.

### Cell counting kit‐8 (CCK-8) assay

HS683 cells and HS683 IDH mutant cells were seeded in 96‐well plates at a density of 10^4 cells per well. The control and siRNA groups of the HS683 cell lines were cultured for 24, 48, and 72 h, respectively, with each group having three duplicate wells. Absorbance at 450 nm was measured after incubation for 1–2 h at 37 °C and 5% CO2.

### Wound-healing assay

After a 6-h period post-transfection, cells underwent harvesting, centrifugation, and resuspension in serum-free culture. Adjusting the cell density to 5 × 10^5^ cells per well, a scratch was introduced into the cell layers when they reached 90% confluence using a 200 μl sterile pipette tip. Following the removal of the cell culture medium, suspended cells, and debris, each well was refilled with serum-free medium and left to incubate for 24–48 h. Subsequent observations and photographic documentation focused on the cell migration area. The scratch test was then employed to evaluate differences in cell healing ability based on the migration area.

### Transwell assay

Matrigel (Corning, USA) was thawed overnight at 4 °C. Subsequently, 100 μl of diluted Matrigel was added to the chamber. The upper chamber received 200 μl of serum-free medium, while the lower chambers were supplemented with 500 μl of 10% FBS DMEM. A total of 3 × 10^4^ harvested cells were seeded in the upper chambers and incubated for an additional 48 h. Following incubation, the invading chamber was removed, and cells on the polycarbonate membrane were fixed with 4% paraformaldehyde and stained with 0.1% crystal violet. Three random fields were selected, and the count of invaded cells was conducted under a microscope. The experiments were replicated in triplicate.

### Statistical analysis

All data are completed by GraphPad Prism 5 software and R software (version 4.2.0). Differential expression was evaluated using the Wilcoxon signed-rank test. We use Student's t-test to analyze continuous variables. The statistically significant cut-off was set at 0.05.

### Institutional review board statement

This study strictly followed the principles of the Helsinki Declaration, and all normal and glioma samples used in the study were approved by the Ethics Committee of Central South Hospital of Wuhan University (No. 2019048).

### Informed consent statement

Patients who provided samples have signed the informed consent form.

## Results

### Stem cell landscape and characteristics of LGG

**Figure 1:**
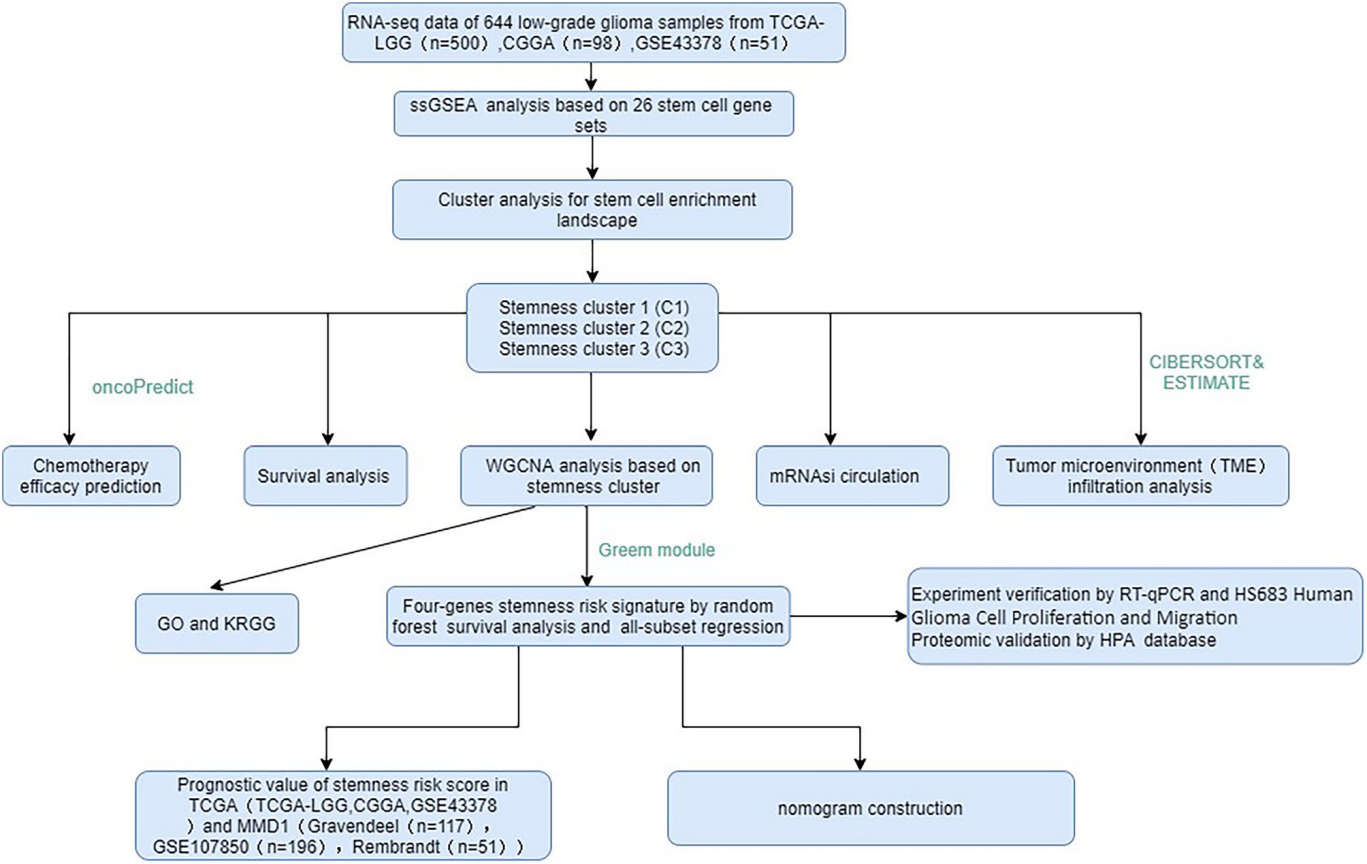
Flow chart of the experiment.

**Figure 2:**
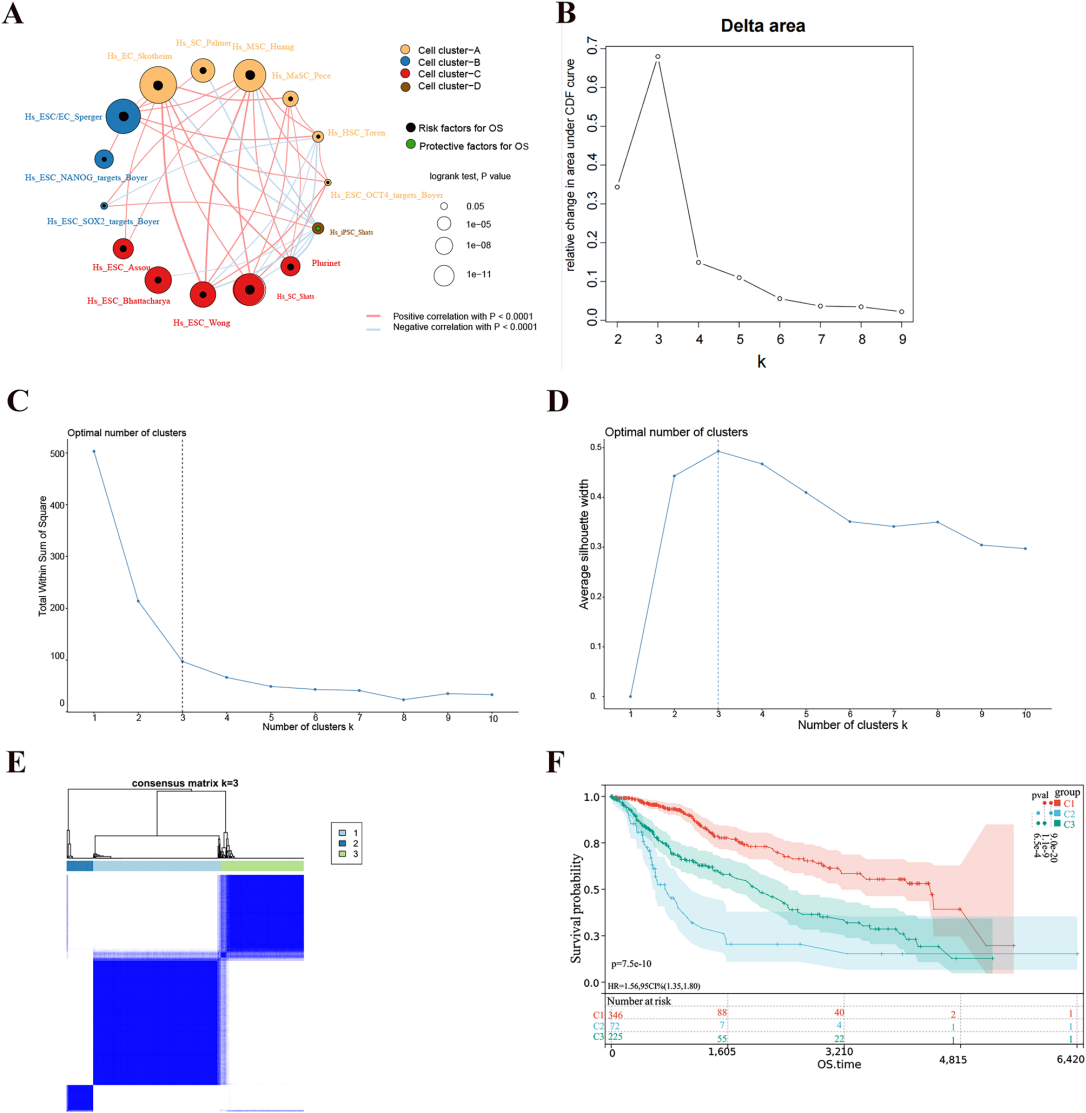
(**A**) Interaction network of 15 prognostic stem cell gene sets, The thicker the line, the stronger the Spearman correlation between gene sets. (**B**) Delta area plot of cluster stability. For each K, the relative change in area under the CDF curve compared to K-1 is calculated, and the point with the slowest rate of area increase is selected as the best K. (**C**) Sum of squared error (wss) plot of the cluster, when the fall of wss suddenly slowers down, the K = 3 is the best K. (**D**) Average silhouette plot. The K = 3 with the largest Average silhouette is the best K. (**E**) Heat map of the consistency matrix at k = 3, where the less white color blocks contain blue, the better the surface clustering effect. (**F**) Kaplan–Meier curves for LGG samples between three clusters.

**Figure 3:**
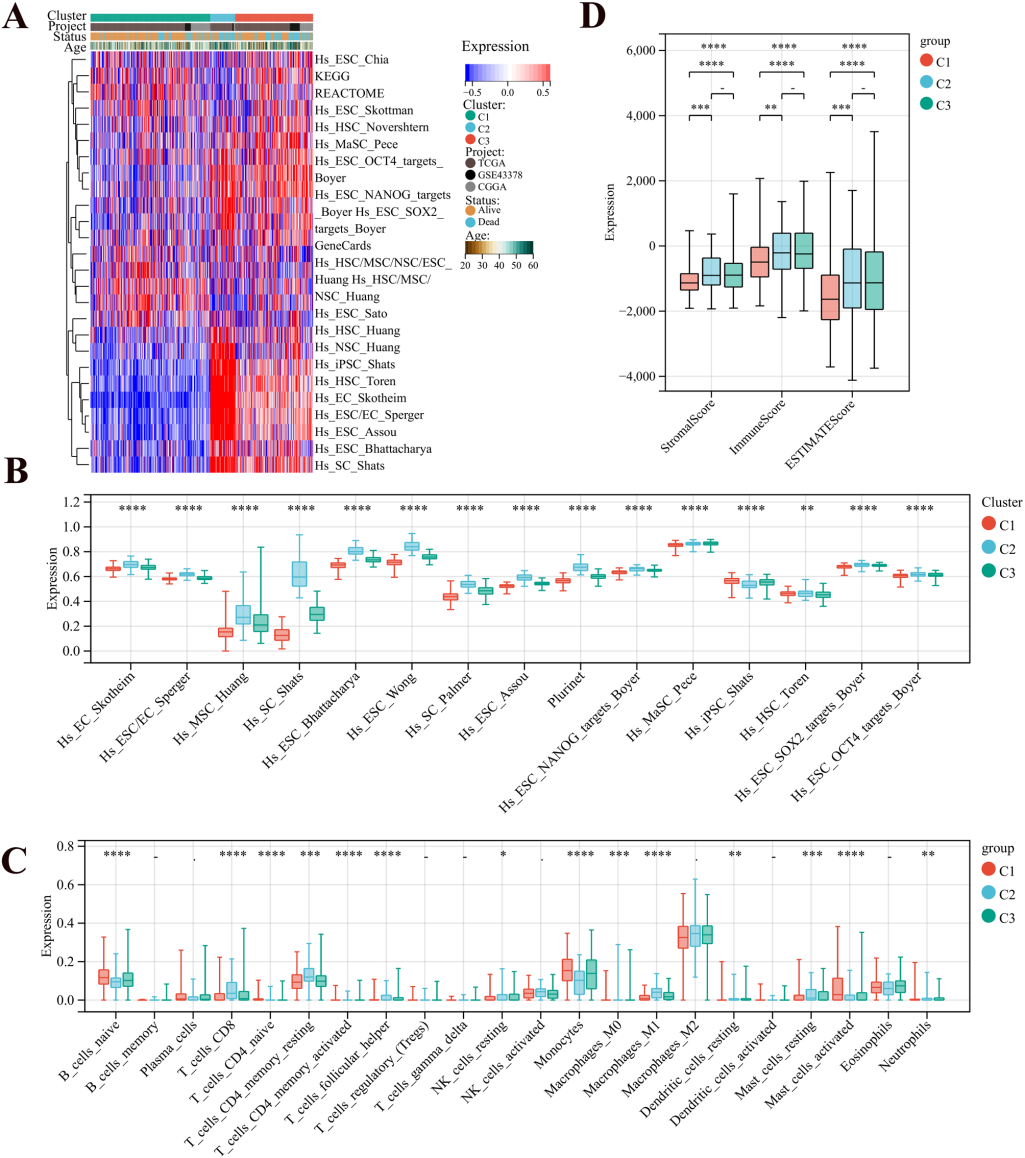
(**A**) Heat map of the enrichment scores for different gene sets. (**B**) Box plots of the enrichment levels of the prognostic gene sets for the three phenotypes. (**C**) Box plots of ESTIMATE scores for the three phenotypes. (**D**) Box plots of cibersort scores for the three phenotypes.

Figure 1 reflects the process of this article, illustrating the steps we took to construct and validate the stem cell-related risk prediction model and the associated influencing factors. In this study, we developed patterns of stemness enrichment and constructed signatures indicating the association between stemness and risk (Fig. 1). The scores reflecting the enrichment related to stemness in LGG samples were quantified using the ssGSEA algorithm, and 15 gene sets were screened out using univariate Cox analysis (P < 0.05) to depict a prognostic stemness network. Among the above 15 gene sets, all but Hs iPsc Shats are risk factors for OS (Fig. 2A). And LGG patients were categorized into three distinct clusters (Fig. 2B–E). Cluster 2 LGG patients had worse OS, whereas Cluster 1 LGG patients had the best OS (log-rank P = 7.5e−10, Fig. 2F). And Cluster2 was enriched by stemness gene sets, which were found to decrease patients' OS, such as Hs SC Shats, Hs SC Palmer, and HS ESC Bhattacharya. The enrichment of all gene sets with prognostic significance in C1 is lower than that in C2, except for the protective gene set Hs iPsc Shats (Fig. 3A,B). This observation further corroborates the earlier conclusion.

Additional understanding of the TME landscape was gained ESTIMATE and CIBERSORT analyses, comparing TME components and stromal and immune scores (Fig. 3C,D). Within the three stemness clusters, Cluster 2 demonstrated an immunosuppressive subtype marked by elevated levels of macrophages M1 and activated mast cells, along with increased immune and stromal scores. On the other hand, Cluster 1 displayed higher antitumor TME components, such as B cells naive and plasma cells, and lower immune and stromal scores.

### Molecular characteristics, sensitivity to chemotherapy and responsiveness to immunotherapy vary across stemness subtypes

**Figure 4:**
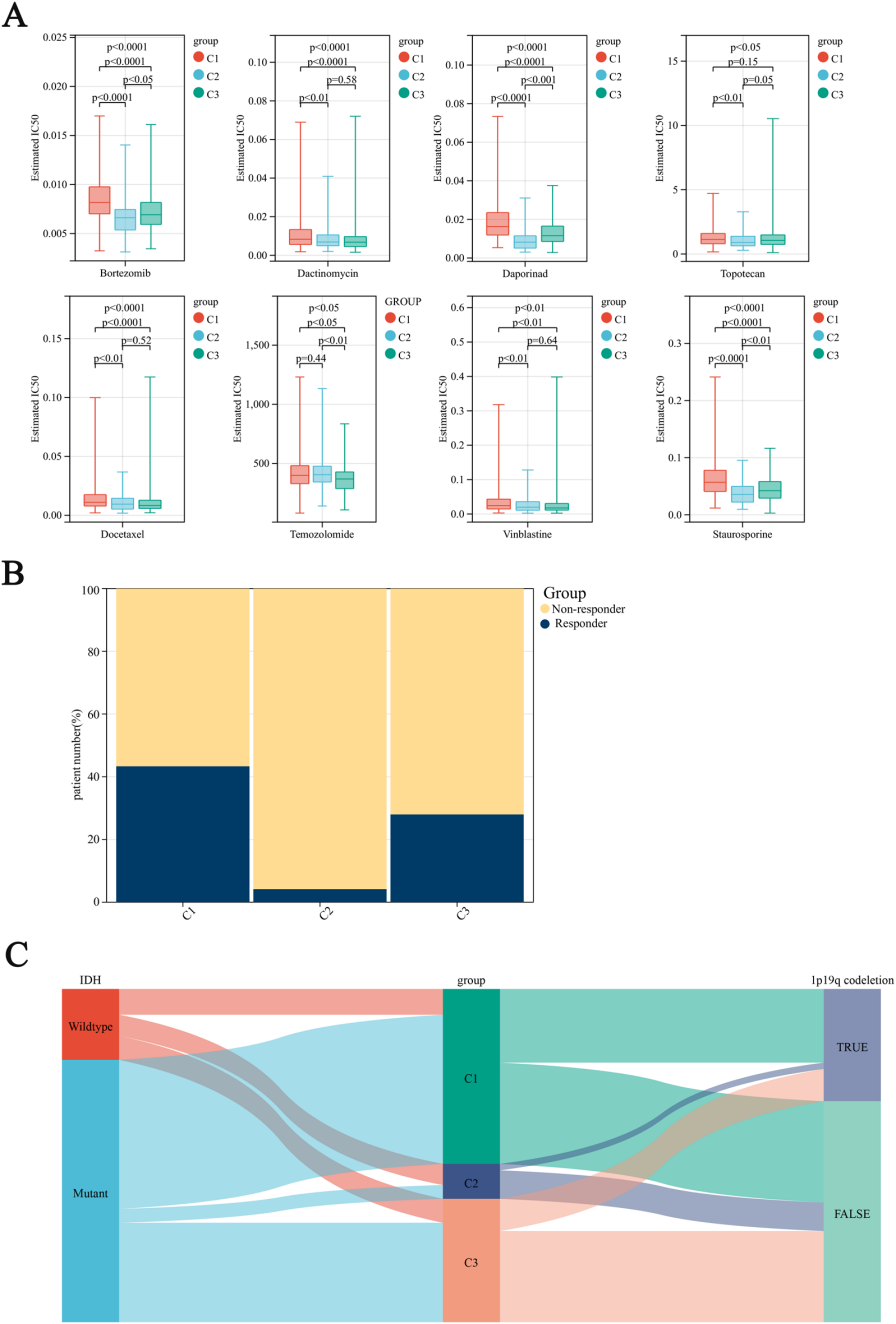
(**A**) Box line plot of IC50 values of chemotherapeutic agents between different stem cell subtypes. (**B**) Estimating the distribution of immunotherapy non-esponders and responders across three phenotypes using the TIDE algorithm. (**C**) Alluvial diagram of three clusters, IDH and 1p19q codeletion.

Currently, the conventional treatment strategy for colorectal cancer patients is surgery plus systemic chemotherapy. To evaluate chemosensitivity, we used a prediction algorithm to estimate the IC50 value of several chemotherapeutic drugs and compared them among stem clusters. As shown in Fig. 4A, the IC50 estimates of Bortezomab, Daporinad, Topotecan, and Staurosporine in Cluster 2 were significantly lower, indicating that this subtype may be more sensitive to these drugs. Additionally, Cluster 3 was found to be more sensitive to temozolomide. Using the TIDE algorithm, we evaluated the immune response of the three subtypes. As the TME environment results suggest, Cluster 1 had higher levels of B cells and other killer cell infiltration with a significantly higher proportion of benefits from immunotherapy than the other clusters. However, the activation of immunosuppressive mast cells and the decrease of M1 macrophages and CD8 T cells may be the reasons for the highest non-response ratio in Cluster 2. As shown in Fig. 4C, the Cluster 1, which has the best prognosis, mostly consists of IDH-mutant types, whereas the Cluster 2, which has the worst prognosis, primarily consists of IDH-wildtype. This is consistent with previous research findings. Similarly, the 1p19q co-deletion, which often indicates a better prognosis, is also mostly found in the Cluster 1. In contrast, the Cluster 2, which has the worst prognosis, only rarely exhibits the 1p19q co-deletion.

### Utilizing WGCNA to identify the module and hub genes associated with stemness Cluster

**Figure 5:**
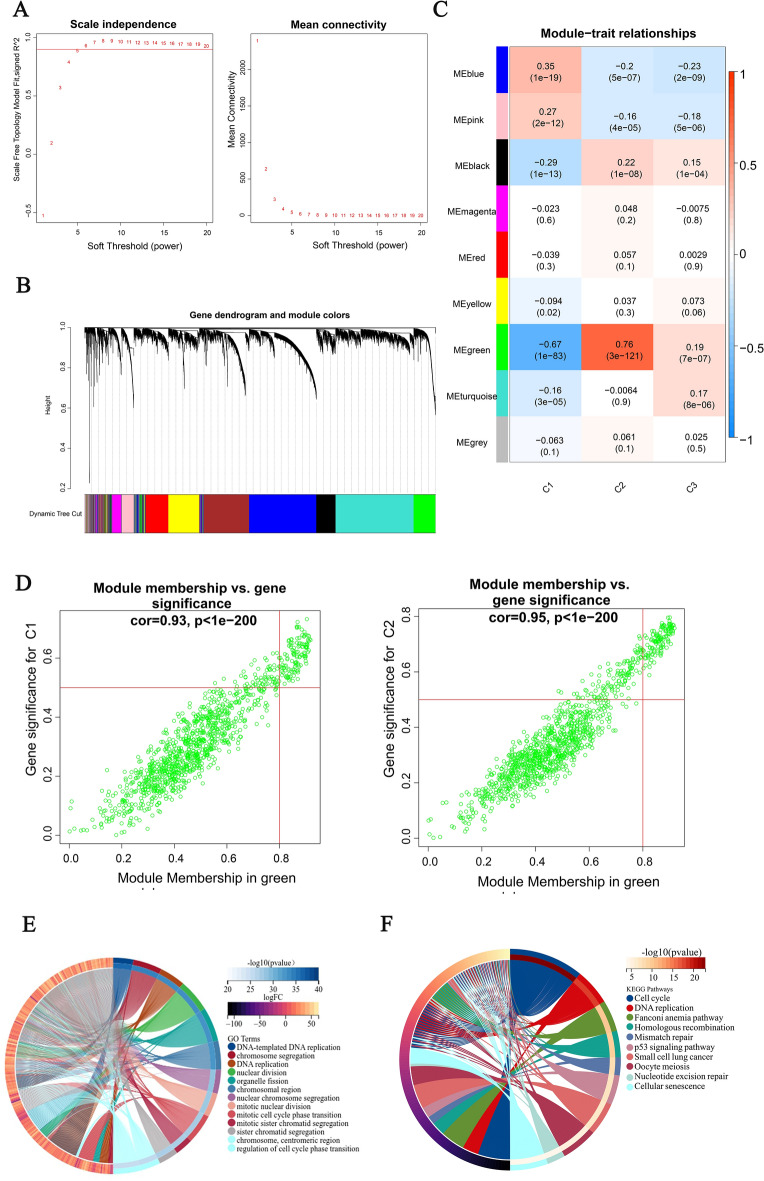
(**A**) Schematic diagram of the WCNGA soft threshold, scale-free topology check when R2 = 5. (**B**) The cluster dendrogram of differentially expressed gene groups. (**C**) Relevance heat map of gene modules and three clusters. (**D**) Scatter plots illustrating the correlation between module membership and gene significance within the green modules. (**E**) GO analysis for genes in green modules. (**F**) KEGG circle diagram for genes in green modules, the width of the color block reflects the importance of the channel.

The difference in immune therapy response and survival outcomes between the C1 and C2 groups of LGG patients is the greatest. To identify characteristic genes of this subtype in TCGA, we used WGCNA analysis. The optimal soft threshold power β was determined as 5, ensuring scale-free network constructions with an unscaled R2 value of 0.9 (shown in Fig. 5A).

We set a minimum of 300 genes for each module and observed that Eight modules were formed by genes with comparable expression profiles with a clustering dendrogram (Fig. 5B). Among these modules, The green module exhibits a robust positive correlation with gene expression in the Cluster 2 subtype (ME = 0.76, P = 3e−121), while demonstrating the strongest negative correlation with the Cluster 1 subtype. (ME =  − 0.67, P = 1e−83) (Fig. 5C). The green module was determined to be the central node and identified 1123 Key genes for subsequent exploration, on the basis of qualification standards of GS > 0.4 and MM > 0.8 (Fig. 5D).

And the GO analysis showed enrichment in DNA-templated DNA replication, chromosome segregation, and DNA replication. Additionally (Fig. 5E), the KEGG analysis revealed that the turquoise module was mainly involved in cell cycle regulation, DNA replication, and the Fanconi anemia pathway (Fig. 5F).

### Constructing a prognostic stemness signature and validation

**Figure 6:**
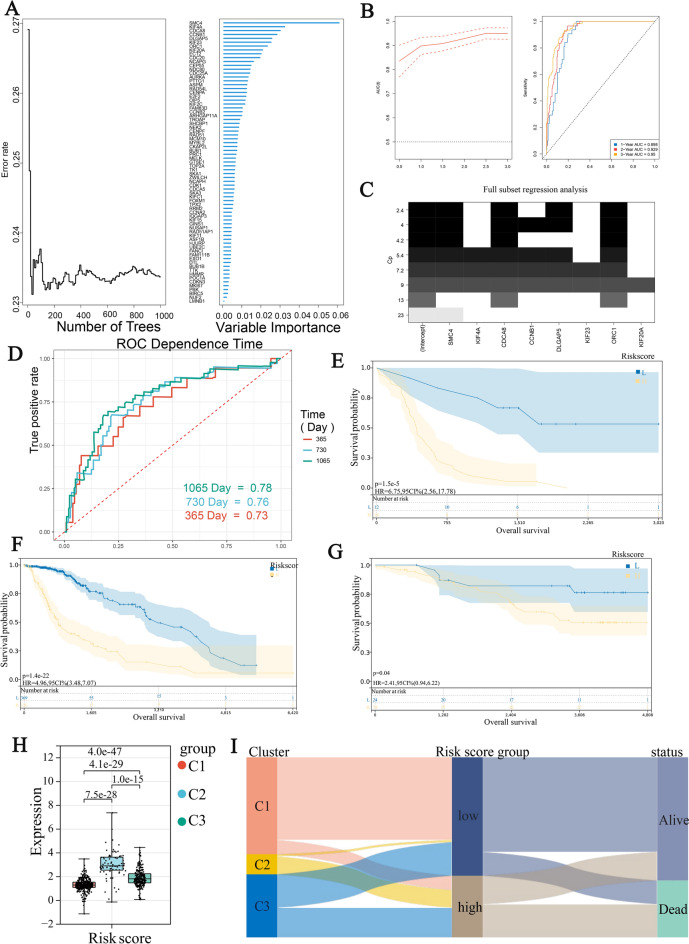
(**A**) Random Forest analysis results of genes screened by single factor Cox analysis, The higher the blue bar, the more important the gene is for OS. (**B**) AUC curves for random forest results. (**C**) Mallower's CP diagram for full subset regression, the four genes with the lowest Mallower's CP were selected for prognostic modeling, Mallower's CP value less than the number of independent variables plus one indicates that the model has better predictive power and less complexity. (**D**) ROC curve in MMD1, AUC > 0.75 proved that this prognostic model is meaningful in the MMD1 data set. (**E**–**G**) Kaplan–Meier analysis for three data sets in TCGA (**H**) Prognostic risk box plot for the three phenotypes (**I**) Alluvial diagram of three clusters, risk score and OS.

We performed a single Cox regression analysis and identified 73 genes that were substantially linked with OS (P < 0.05). Next, we filtered out genes that were not important for OS using random forest survival analysis. resulting in the selection of 8 genes with relative importance > 0.25 (Fig. 6A). The ROC curve showed that the number of the area under the curve (AUC) in three years was greater than 0.89, which indicated the high accuracy of the selected genes as prognostic markers (Fig. 6B).

The 8 genes were assembled into a set for analysis, and Fig. 6C shows that SMC4, CDCA8, DLGAP5, and ORC1 were selected. We developed this formula to calculate the risk value for each sample: Risk score = (0.065 * expression of SMC4) + (0.040 * expression of CDCA8) + (− 0.032 * expression of DLGAP5) + (− 0.047 * expression of ORC1). Using this score, the stemness-risk score was calculated for each LGG patient. The ROC curve analysis showed that 1-year AUC = 0.73, 2-year AUC = 0.76, and 3-year AUC = 0.78 (MMD1) (Fig. 6D).

The stemness-risk signature's ability to predict overall survival (OS) was validated in other cohorts, including CGGA, TCGA-LGG, and GSE43378. The Kaplan–Meier survival curves demonstrated that patients with high stemness-risk scores had shorter OS time compared to those with lower stemness-risk scores (CGGA: log-rank test, P = 1.5E-5; TCGA-LGG: P = 1.4E−22; GSE43378: log-rank test, P = 0.04) (Fig. 6E–G). Stemness cluster C2 had the highest stemness-risk score, while cluster C1 had the lowest (Fig. 6H). The relationship between cluster, each group's stemness risk score, and status was consistent with previous findings (Fig. 6I).

### Correlation between risk characteristics and tumor microenvironment

**Figure 7:**
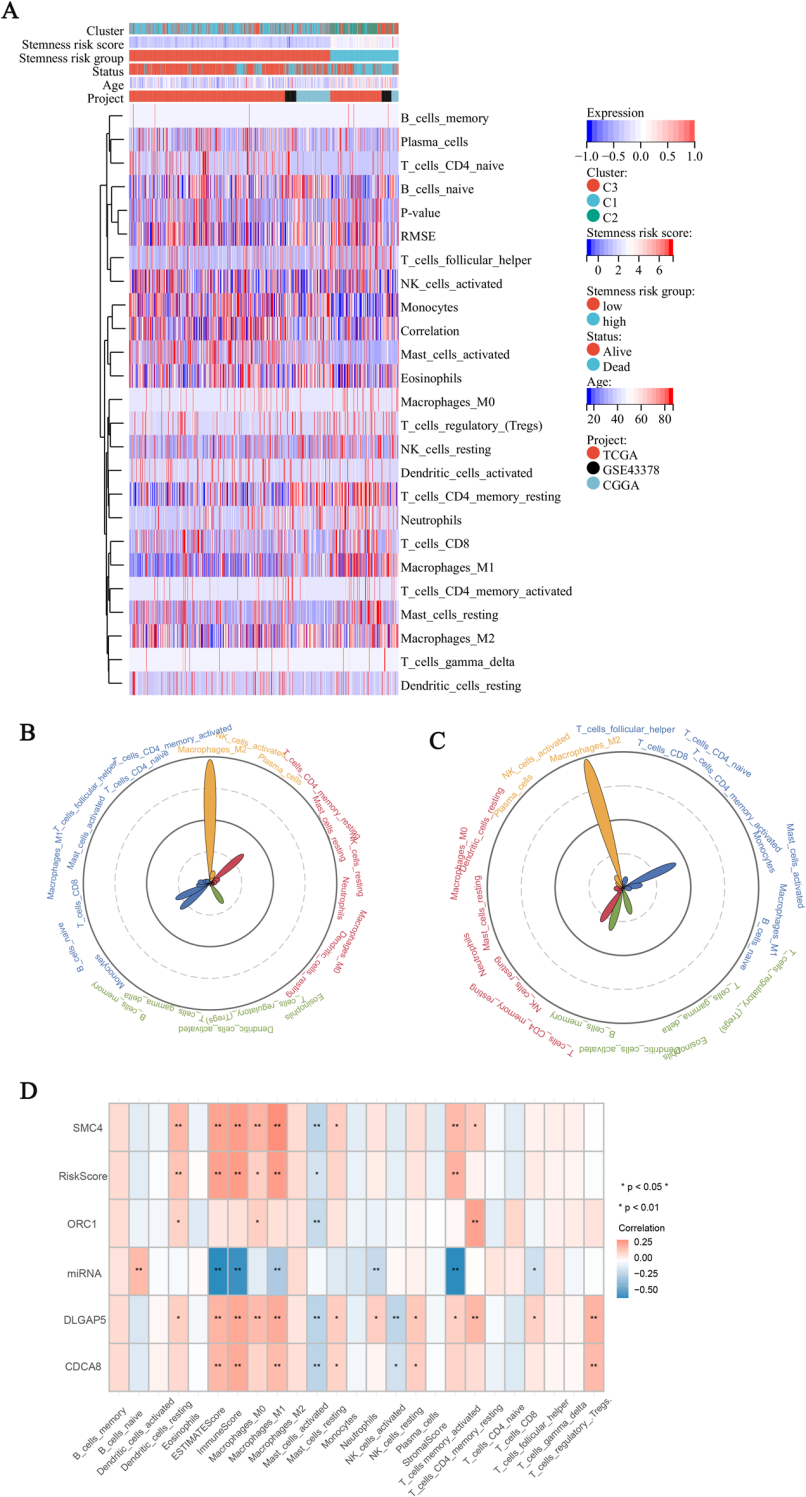
(**A**) Heat map reflecting the degree of distribution of immune cells in samples of different risk score groups. (**B**) Spearman correlation diagram of factors of risk score, hub genes and mRNAsi. (**C**,**D**) Radar plot showing the extent of immune cell enrichment in the lower-risk group (**C**) and high-risk group (**D**).

The heatmap (Fig. 7A) and Spearman correlation diagram (Fig. 7B) showed that macrophage B cells naive, monocyte and mast cell activated had a higher degree in the lower-risk group, indicating that this group may be more responsive to immunotherapy. Conversely, the high stemness-risk group was influenced by T cell CD4 memory rest, M2 macrophages, and M1 macrophages, indicating that immune function was suppressed. The immune score was significantly higher in the high-risk group compared to the lower-risk group. Figure 7C and D show the mean distribution of TME in the two groups. The most important immune response in the lower stemness risk group was T-cell regulation, suggesting differentiation of immune function toward antitumor, while the high stemness risk group had higher levels of immunosuppressive cells, especially M2 macrophages.

### Confirmation of the expression of hub genes

**Figure 8:**
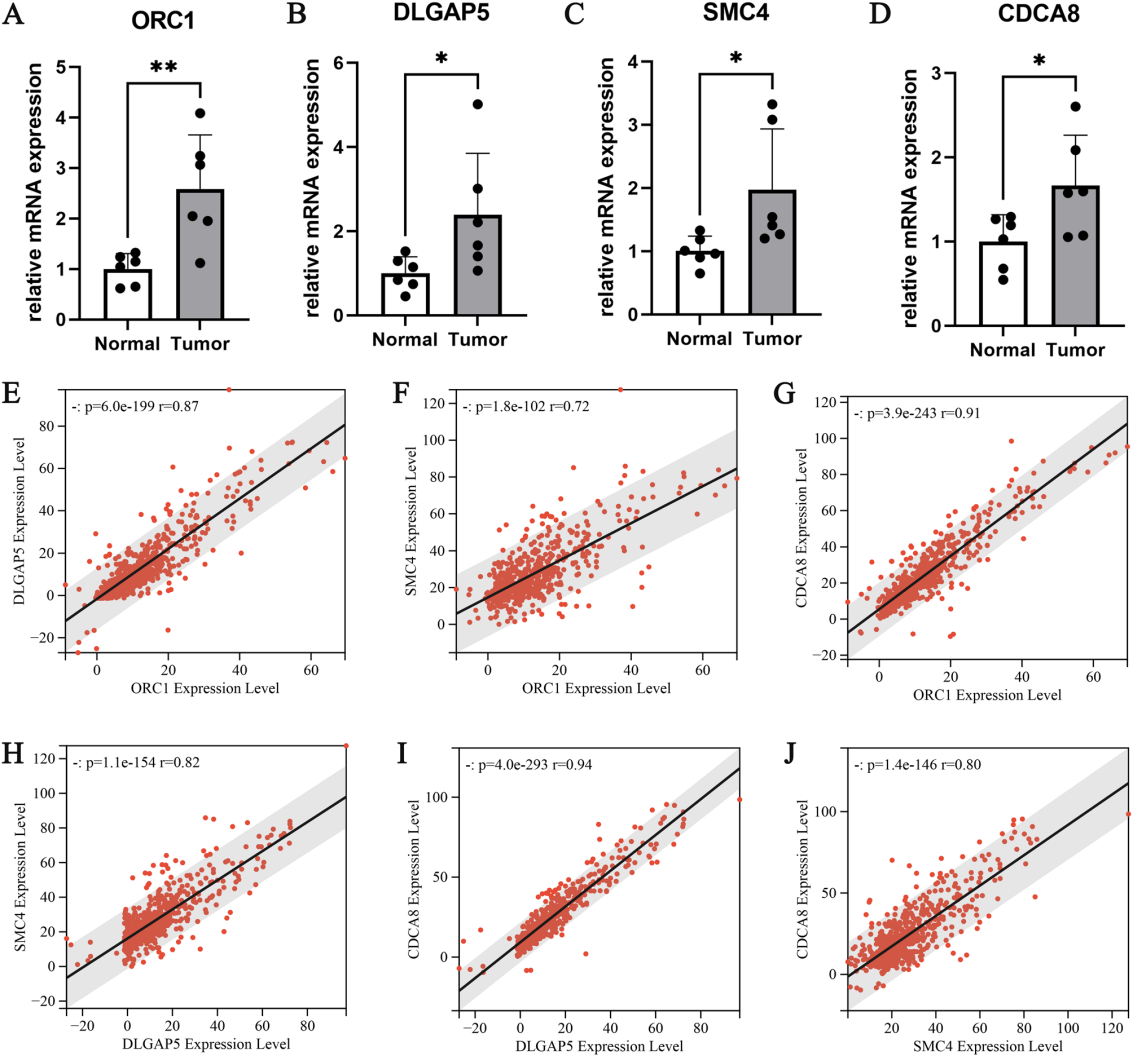
qPCR results of four hub genes: (**A**) ORC1, (**B**) DLGAP5, (**C**) SMC4 and (**D**) CDCA8. Expression correlation between hub genes: (**E**) ORC1 and DLGAP5, (**F**) ORC1 and SMC4, (**G**) ORC1 and CDCA8, (**H**) DLGAP5 and SMC4, (**I**) DLGAP5 and CDCA8, (**J**) SMC4 and CDCA8.*p < 0.05, **p < 0.01, ***p < 0.001, ****p < 0.0001; ns, no significance.

**Figure 9:**
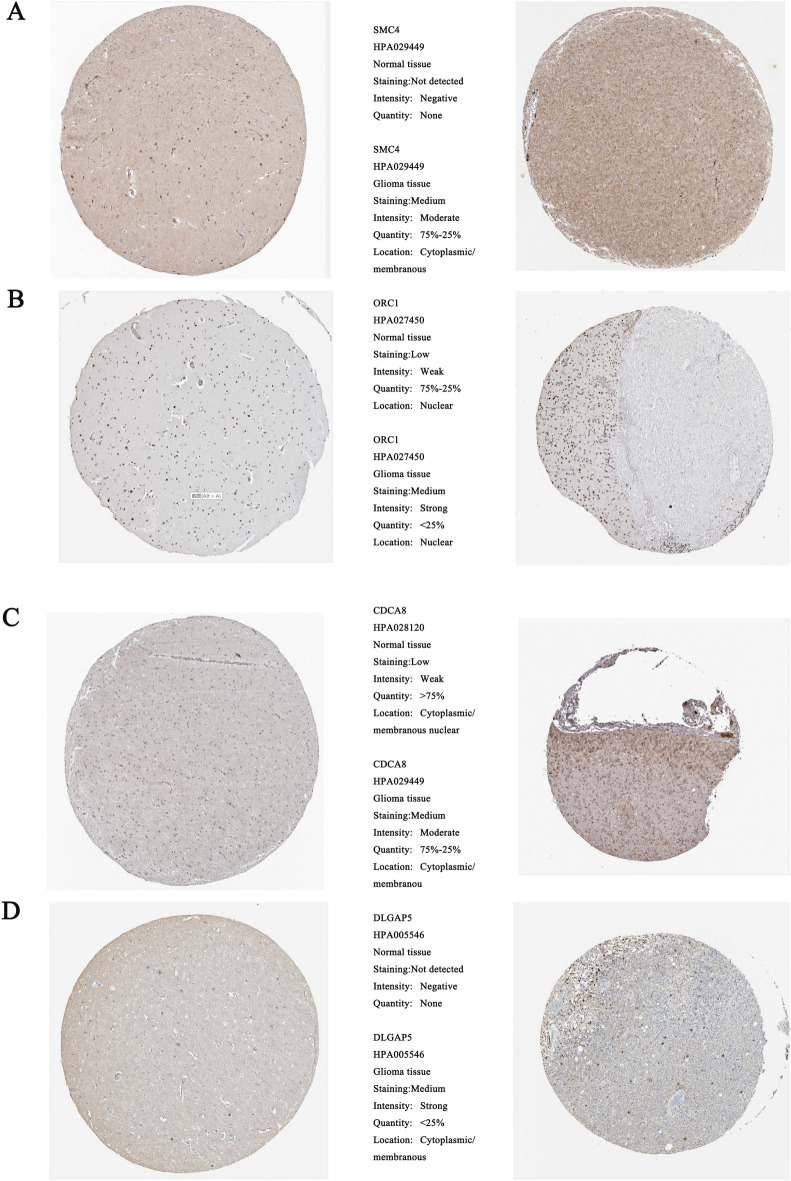
Comparison of normal tissue expression and LGG tissue expression of the four key genes in the HPA database: (**A**) SMC4, (**B**) ORC1, (**C**) CDCA8 and (**D**) DLGAP5.

The results of qPCR indicated that OCR1 (P < 0.05; Fig. 8A), DLGAP5 (P < 0.05; Fig. 8B), SMC4 (P < 0.05; Fig. 8C), and CDCA8 (P < 0.05; Fig. 8D) , compared to normal tissue, it is highly expressed in glioma tissue. We also investigated the Spearman correlation of mRNA expression levels for the four hub genes. We found that all four genes are strongly positively correlated, which is consistent with our research findings(Fig. 8E–J) . Furthermore, we confirmed the translation expression level of the hub genes using the HPA database, and the prognostic biomarkers were found to be stained strongly or moderately.: SMC4 (Fig. 9A), OCR1 (Fig. 9B), CDCA8 (Fig. 9C), and DLGAP5 (Fig. 9D), indicating that these hub genes were translated more in glioma samples.

### Investigating the impact of hub genes on HS683 human glioma cell proliferation and migration

**Figure 10:**
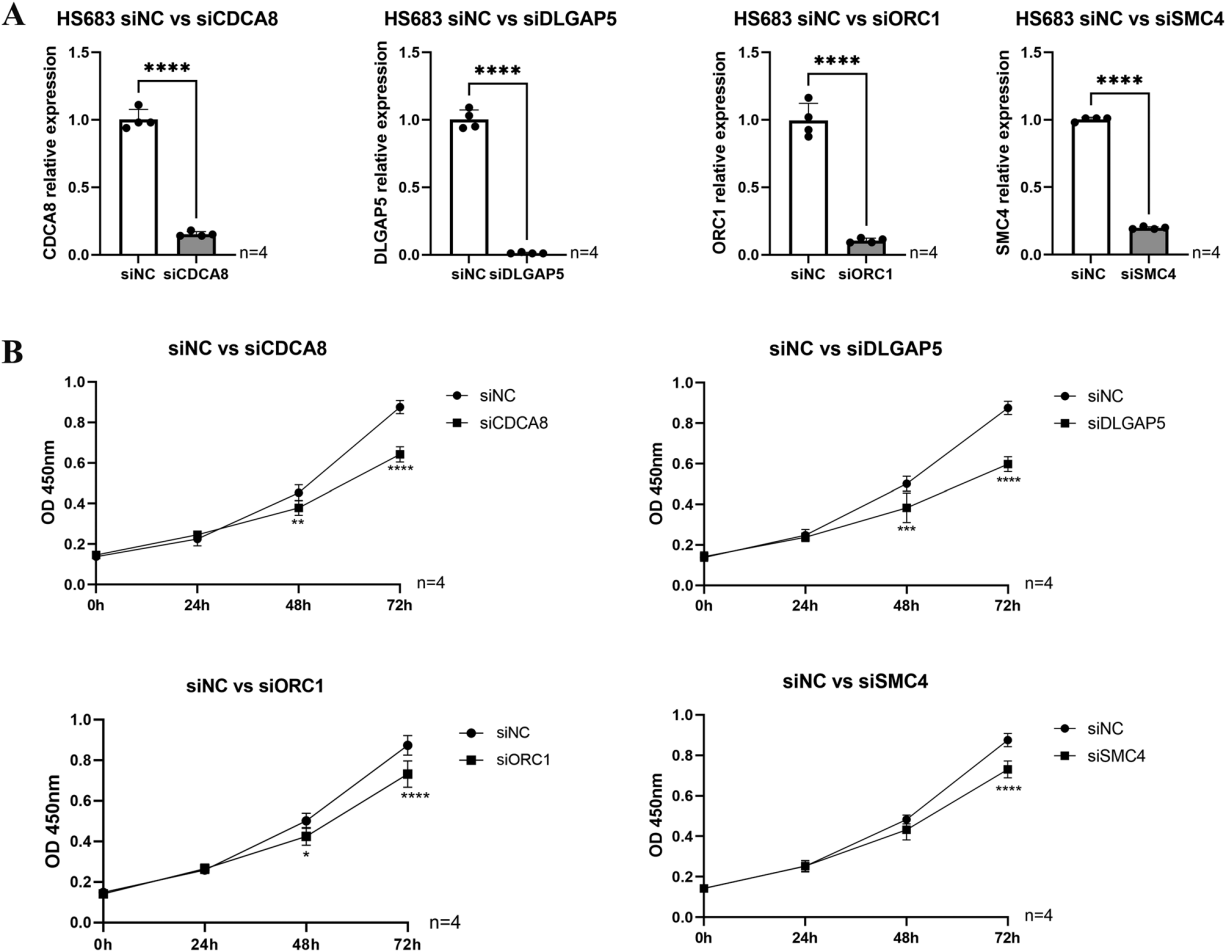
(**A**) The qRT-PCR assays validated the siRNA knockdown effect. (**B**) The results of CCK-8 assay in HS683 cell lines. *p < 0.05, **p < 0.01, ***p < 0.001, ****p < 0.0001; ns, no significance.

**Figure 11:**
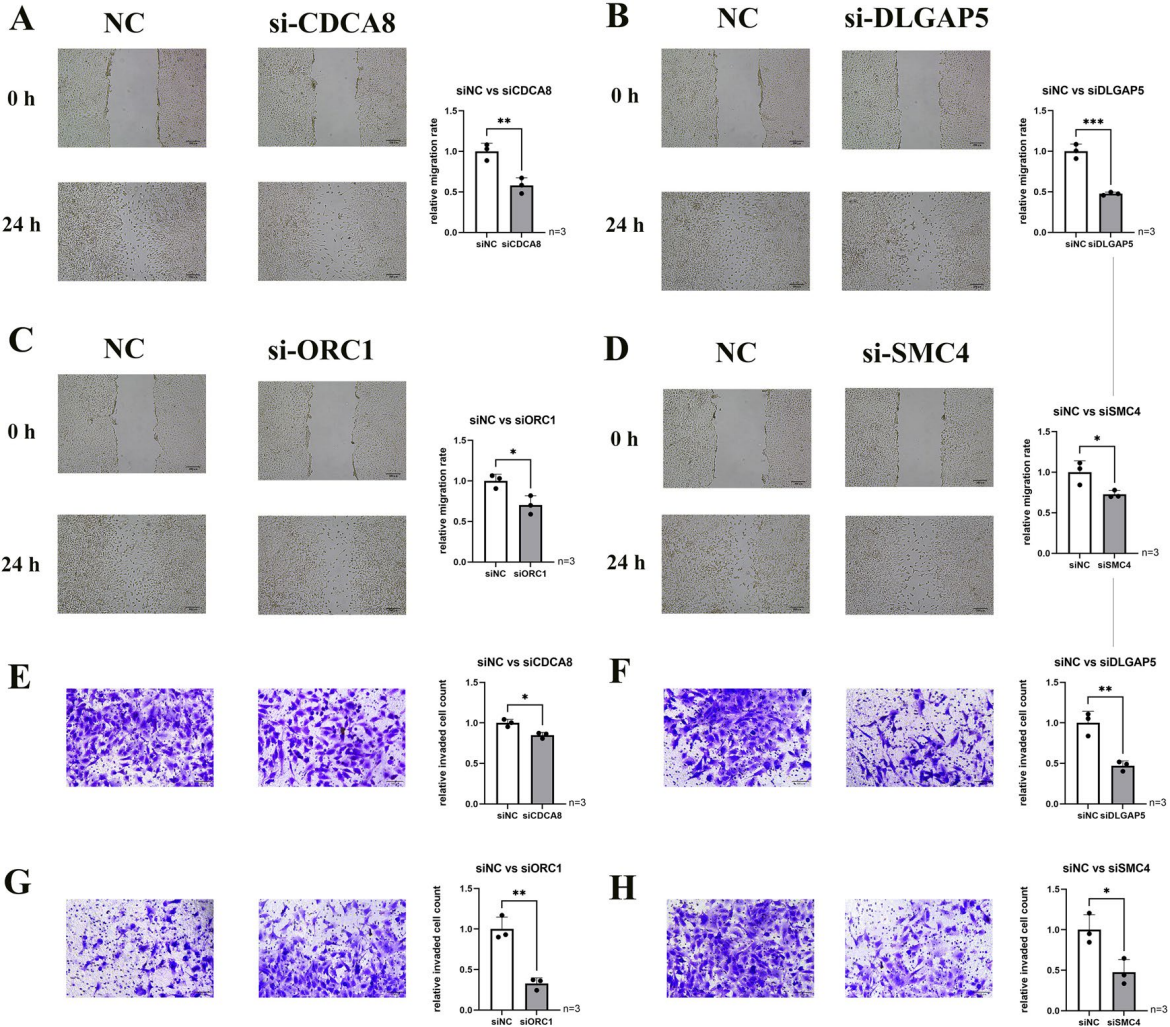
Scratch wound healing assessment of HS683 cell lines following treatment with siRNA or negative control (NC) targeting four hub genes: (**A**) CDCA8, (**B**) DLGAP5, (**C**) ORC1 and (**D**) SMC4. Transwell examination of LGG cell lines treated with siRNA or negative control (NC) to target four hub genes: (**E**) CDCA8, (**F**) DLGAP5, (**G**) ORC1 and (**H**) SMC4. *p < 0.05, **p < 0.01, ***p < 0.001, ****p < 0.0001; ns, no significance.

**Figure 12:**
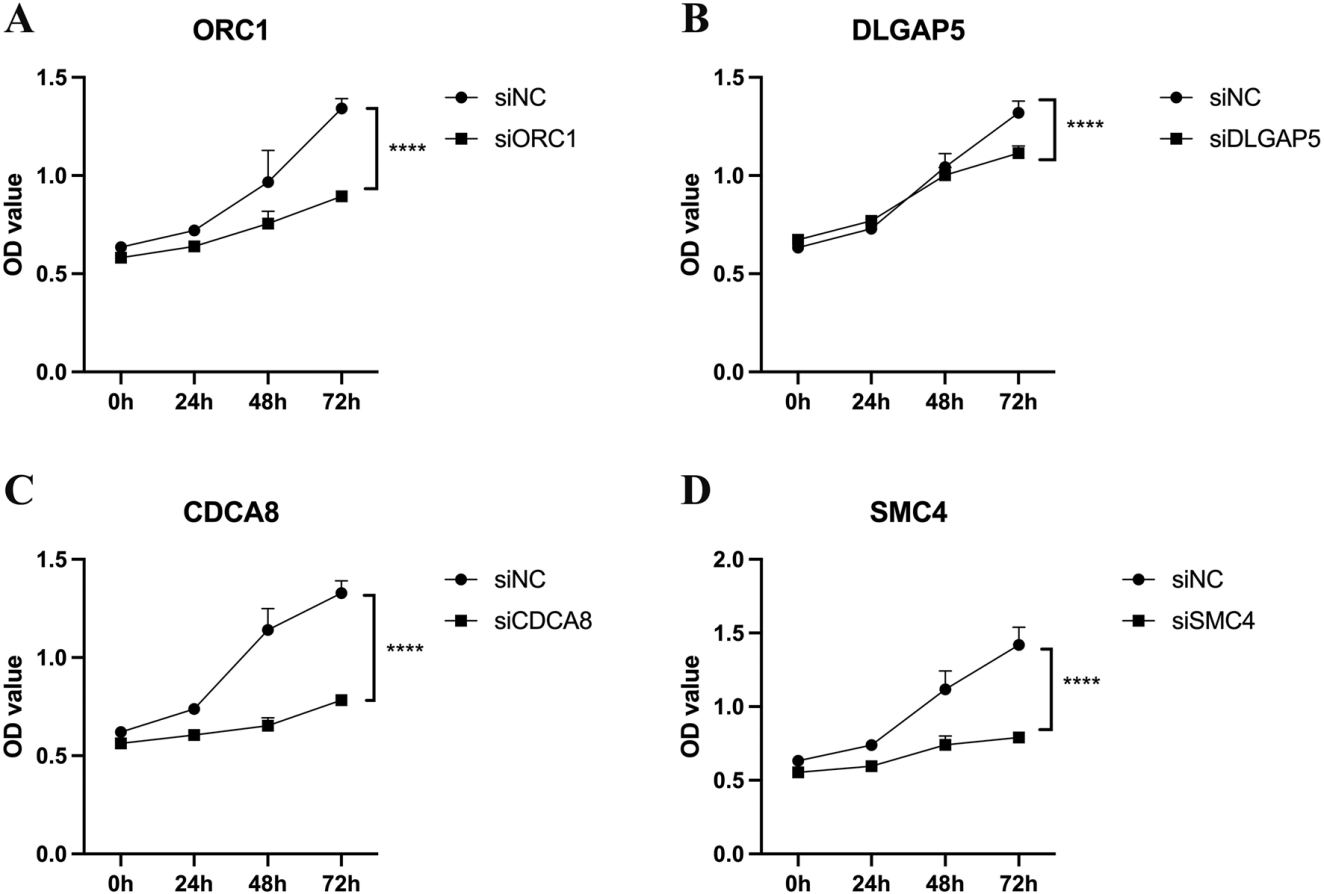
The results of CCK-8 assay in HS683 cell lines. (**A**) SMC4, (**B**) DLGAP5, CDCA8 and (**D**) ORC1. *p < 0.05, **p < 0.01, ***p < 0.001, ****p < 0.0001; ns, no significance.

**Figure 13:**
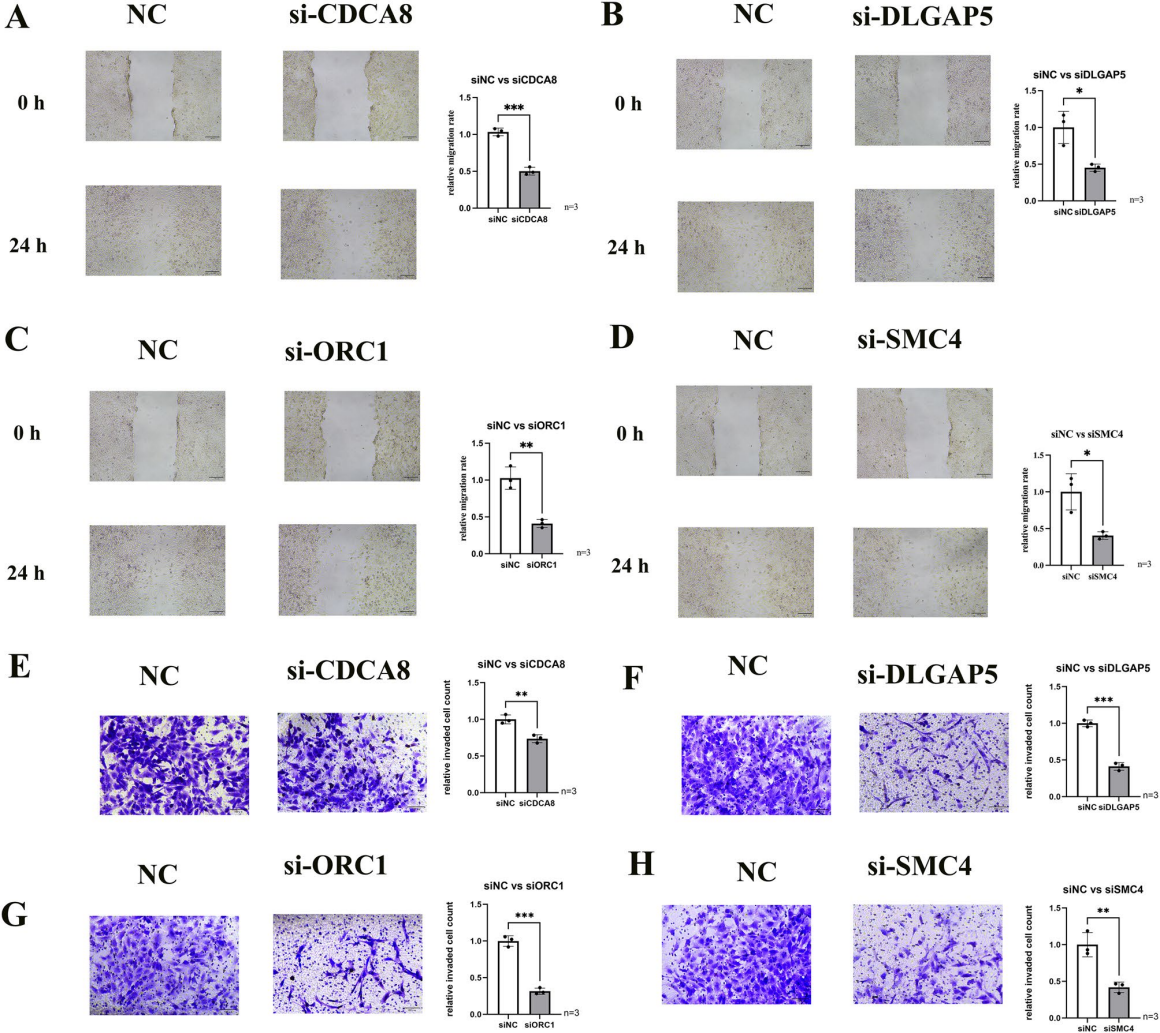
Scratch wound healing assessment of IDH mutant cell lines following treatment with siRNA or negative control (NC) targeting four hub genes: (**A**) CDCA8, (**B**) DLGAP5, (**C**) ORC1 and (**D**) SMC4. Transwell examination of LGG cell lines treated with siRNA or negative control (NC) to target four hub genes: (**E**) CDCA8, (**F**) DLGAP5, (**G**) ORC1 and (**H**) SMC4. *p < 0.05, **p < 0.01, ***p < 0.001, ****p < 0.0001; ns, no significance.

To ascertain the involvement of hub genes in HS683 human glioma cell proliferation and migration, we conducted additional experimental investigations focusing on prognostic markers. Subsequently, we implemented loss-of-function experiments to silence four hub genes in HS683 cells, aiming to elucidate the role of hub genes in LGG progression. The efficacy of siRNA knockdown in HS683 cells was validated using qRT-PCR (Fig. 10A). A significant reduction in cell viability within 72 h was observed through the CCK-8 assay after the knockdown of CDCA8, DLGAP5, ORC1, and SMC4 (Fig. 10B). Furthermore, transwell invasion and scratch assay results demonstrated a substantial decrease in HS683 cell migration and invasion following the knockdown of CDCA8, DLGAP5, ORC1, and SMC4 (Fig. 11). After verifying the significant IDH1 mutation in the HS683 cell line through qPCR,, the aforementioned in vitro experiments were also validated in the IDH mutant HS683 cell line.(Figs. 12, 13). Whether in IDH-mutant HS683 cell lines or IDH-wildtype HS683 cell lines, knocking down the four hub genes significantly reduced the invasive growth capability of glioma cells, further proving that these four genes are oncogenes.

### Clinical application of risk score

**Figure 14:**
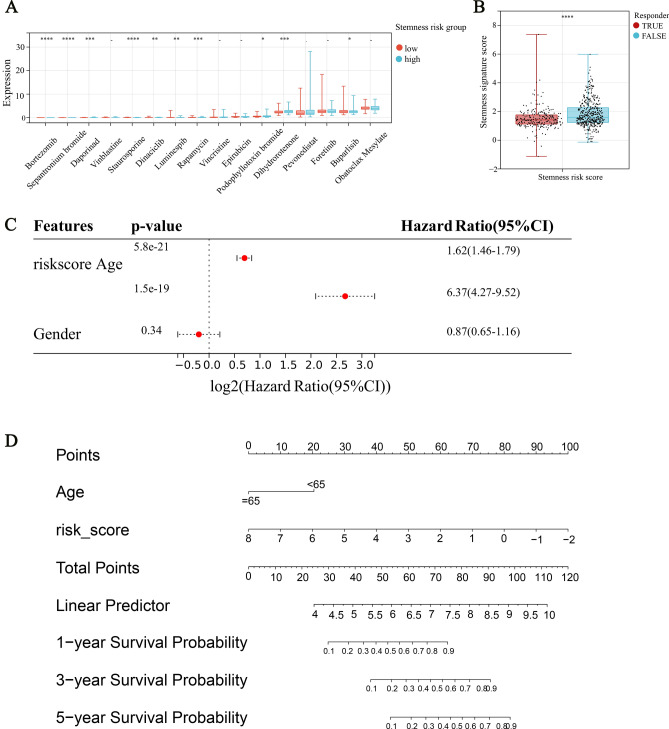
(**A**,**B**) Box plot of chemotherapy (**A**) and immunotherapy response (**B**) in different risk groups. (**C**) The results of univariate analysis of risk score, age and gender based on TCGA. (**D**) A nomogram calculating the patient's OS at 1, 3 or 5 years. The line length reflects the risk level associated with the factor, and the scale enables the evaluation of the risk score.

Our previous findings have indicated that stemness Cluster C2 is more responsive to certain chemical drugs but less responsive to immunotherapy (as shown in Fig. 4B). Additionally, our observations have shown that the high stemness-risk group is more sensitive to drugs that are effective on C2, such as Bortezomab, Daporinad, Topotecan, and Staurosporine (as depicted in Fig. 14A). Afterward, we analyzed the correlation between the immunotherapy response and stemness-risk model using the TIDE analysis. The results shown in Fig. 14B indicate that patients who positively responded to treatment had significantly lower stemness-risk scores compared to non-responders (Wilcoxon test, P < 0.001).

We endeavored to construct a nomogram that would provide doctors with a practical and effective tool for prognosis decision-making. Our univariate Cox analysis revealed that both the risk score (P = 5.8E-21) and age (P = 1.5E-19) were significantly associated with the overall survival (OS) of LGG patients (as illustrated in Fig. 14C). Subsequently, we constructed a nomogram using the stemness risk score and age, as illustrated in Fig. 14D.

## Discussion

For decades, numerous publications have described the interactions of cancer cells and the immune system. This is also applicable to other components of the TME. However, only recently has research begun to reveal the specific relationship between CSCs and Immune cells reflecting the regulation of immune function in the TME, leading to the development of rational therapeutic strategies to exploit the CSC-immune axis. This study presents the first systematic bioinformatics analysis revealing the molecular subtyping of stemness features in a large cohort of LGG patients, discussing the prognosis, TME infiltration patterns, and treatment response of LGG patients with different subtypes, and describing the gene expression associated with CSC subtypes. These findings offer new strategies for guiding more effective patient-specific treatments.

With the unsupervised clustering identification predicated on 26 stemness gene sets' ssGSEA scores for three different stemness subtypes, the C1 subtype is characterized by a high enrichment level of CSCs gene sets which support good prognosis, and an anti-tumor TME pattern such as enriched immature B cells and plasma cell infiltration, which makes it more sensitive to immunotherapy. In contrast, cluster 2 shows a high enrichment of CSCs gene sets which indicates poor prognosis. Cluster C2 showed higher infiltration of M1 and M0 macrophages and stromal scores, indicating that C2 is an immunosuppressive phenotype that may be less responsive to immunotherapy. In order to explore the genomic characteristics of each cell subtype, we conducted a comprehensive WGCNA analysis to screen modules associated with stem cell properties. Among them, the green module has the highest positive correlation with cluster C3 and the highest negative correlation with cluster C2. It is considered the most critical module and will be included in the future study. GO analysis showed that the primary biological process of the green module was the cell cycle. Previous research results show that dysregulation of stem cell cycle regulation may lead to normal tissue carcinogenesis, and in the progression of glioma, disruption of the cell cycle can also lead to the continuous deteriorating of glioma^51,52^. Then, through subset regression and random forest survival analysis, we identified prognostic hub genes within the green module and constructed a four-gene stemness signature based on these hub genes to quantitatively evaluate the prognostic risk of samples. Then we concluded that the C2 stemness subtype exhibited a higher risk score than the other two groups which means a poor prognosis.

Our analysis revealed that LGG patients with lower-risk characteristics have antitumor immunity, high infiltration of monocytes, and both activated and quiescent mast cells. On the other hand, high-risk LGG patients have an abundance of immune-suppressive M1/M2 macrophages and CD4 memory T cells. Among them, M2 macrophage has the highest infiltration content in the high-risk group, indicating their role in enhancing tumor invasiveness and mediating immunosuppression. Impaired CD4+ T effector memory cell function was associated with the proliferation of myeloid-derived suppressor cells (MDSCs) in glioma patients^53^. MDSCs can suppress other immune cells in the tumor microenvironment by inducing the expression of microRNA-101 to promote the CSC phenotype^54^. glioma CSCs produce cytokines, including colony-stimulating factor, TGFβ, and macrophage-inhibitory cytokine, to promote M2 macrophage polarization and MDSC recruitment^55–57^, leading to immune suppression and native M2 phenotype polarization. In conclusion, CSCs promote M2 macrophage polarization and MDSC recruitment by affecting the normal expression of MHC-I molecule and the release of immune-suppressive cytokines, facilitating the formation of a tumor immune-suppressive microenvironment^58^.

In terms of chemotherapy, in addition to temozolomide, which is commonly used for glioma patients^59^, we have found that several drugs, including bortezomib, daporinad, topotecan, and staurosporine, show sensitivity to high-risk gliomas. If delivered directly into the tumor through infusion, they will be delivered directly to the brain tumor, providing multiple possibilities for the effective treatment of glioma^60^. In terms of immunotherapy guidance, we conducted TIDE analysis, and LGG patients with lower stemness risk scores often have higher immune therapy responses. This confirms the predictive validity of our stemness model.

The OCLR method, developed for the mRNAsi using datasets of pluripotent stem cells and their progenitor cells^61,62^, plays a crucial role in our analysis. This index evaluates the activity of CSCs and malignant cell dedifferentiation in approximately 644 LGG samples from TCGA^63^. Similar to previous research results, our research shows that LGG patients who had higher mRNAsi values have better prognoses^64,65^ due to their strong abilities of self-renewal, metastasis, and treatment resistance, supported by increasing evidence. However, there is considerable heterogeneity in the markers and models of CSCs among different cancers^66^. In our study, we utilized a multitude of CSC gene sets to identify three distinct stemness clusters. We subsequently developed a stemness-related prognostic model that exhibited a strong negative correlation with mRNAsi, with a correlation coefficient of -0.71. We also observed that the high-risk stemness group was enriched with markers related to the cell cycle, oxidative phosphorylation, local adhesion, pancreatic cancer, homologous recombination, and progesterone-mediated cancer cell maturation, as determined by GSEA analysis. Interestingly, defects in the control of the cell cycle can lead to tumorigenesis through metabolic remodeling and immune escape.^67–69^.

Of the four stemness model genes identified in this study, SMC4 regulates the cell cycle and impacts the proliferation, migration, and invasion of glioma^70,71^. CDCA8 is a cell cycle regulator and tumor promoter that produces a marked effect in various malignant tumors^72^. For example, it regulates the migration of glioma cells and inhibits cell apoptosis^73^. Knockdown of DLGAP5 can significantly inhibit cell proliferation and simultaneously induce G2/M phase cell cycle arrest and cell apoptosis^74^. Chen et al. reported that tumor samples with lower expression of ORC1 would reduce the expression of Bcl-2, block the cell cycle, and increase the apoptosis rate^75^.

This study has some limitations. Although it is based on multiple datasets, it lacks further experimental verification and detailed data on immunotherapy and chemotherapy. Follow-up studies should carry out more experiments to clarify the potential molecular mechanism of stem cells acting on LGG. Furthermore, we need to use more of our clinical data to verify it soon. The specific mechanism target of CSCs is not clear, and further mechanism-related experiments are necessary to verify the TME-related conclusions to better serve the treatment progress of LGG.

## Conclusion

Our study illuminates the stemness characteristics of gliomas, which lays the foundation for developing therapeutic approaches targeting CSCs and enhancing the efficacy of current immunotherapies. By identifying the stemness subtype and its correlation with prognosis and TME patterns in glioma patients, we aim to advance the development of personalized treatments, enhancing the ability to predict and improve overall patient prognosis.

### Supplementary Information

Supplementary Information.

## Data Availability

All the website addresses of the databases used in this article are provided in the text.
