# Supplementary material for: Stemness subtypes in lower-grade glioma with prognostic biomarkers, tumor microenvironment, and treatment response
Source: Sci Rep. 2024 Jun 26;14:14758. doi: 10.1038/s41598-024-65717-7 (PMC11208487; doi:10.1038/s41598-024-65717-7)

Supplementary Table S1:The primer sequences for four key genes and IDH1.

|  | Forward (5’-3’ sequence) | Reverse (5’-3’ sequence) |
| --- | --- | --- |
| CDCA8 | CTTCGCCCTTGGAGGAAACAA | GGTGTCTGAATAGCTTCTGCTG |
| DLGAP5 | AAGTGGGTCGTTATAGACCTGA | TGCTCGAACATCACTCTCGTTAT |
| SMC4 | GGCTGTATGGGCGAAAAAGAT | TTGTGGCTTGATCCAAGTTGT |
| ORC1 | ACCGAGATTCACATCCAGATTGG | CGAGCACGTTTCTTAGGAGGA |
| IDH1-R132H | GGCTTGTGAGTGGATGGGTA | CCAGGCCCAGGAACAACAAA |

Supplementary Table S2:The siRNA sequences for four key genes.

| siRNA | CDCA8 | GTTTGACTCAAGGGTCTTCAA |
| --- | --- | --- |
| siRNA | DLGAP5 | CGAGAGTGATGTTCGAGCAAT |
| siRNA | ORC1 | GCCAGAGCGAATCATGATGAA |
| siRNA | SMC4 | GCCCAACAAGACAAACTTGAT |

Supplementary Figure S1: qPCR validation of IDH R132 mutation in HS683 cell line. *p < 0.05, **p <0.01, ***p < 0.001, ****p < 0.0001; ns, no significance.


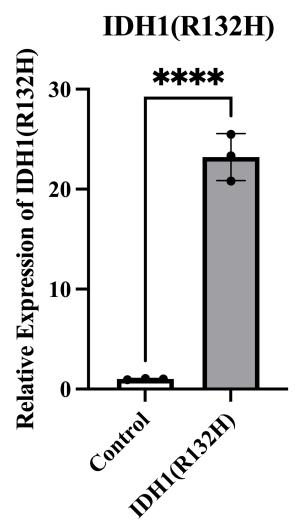

Supplement: Supplementary file 1 — Supplementary Information. [file 41598_2024_65717_MOESM1_ESM.docx]
